# Systematic review of structural interventions for intimate partner violence in low- and middle-income countries: organizing evidence for prevention

**DOI:** 10.1186/s12889-015-2460-4

**Published:** 2015-11-23

**Authors:** Christine Bourey, Whitney Williams, Erin Elizabeth Bernstein, Rob Stephenson

**Affiliations:** Department of Epidemiology, Emory University Rollins School of Public Health, 1518 Clifton Road NE, Atlanta, Georgia 30322 USA; Hubert Department of Global Health, Emory University Rollins School of Public Health, 1518 Clifton Road NE, Atlanta, Georgia 30322 USA; Department of Health Behavior and Biological Sciences, University of Michigan School of Nursing and Center for Sexuality and Health Disparities, 400 North Ingalls, Ann Arbor, Michigan 48109 USA

**Keywords:** Intimate partner violence, Prevention, Structural intervention, Low- and middle-income countries (LMIC), Systematic review

## Abstract

**Background:**

Despite growing attention to intimate partner violence (IPV) globally, systematic evaluation of evidence for IPV prevention remains limited. This particularly is true in relation to low- and middle-income countries (LMIC), where researchers often organize evidence by current interventions strategies rather than comprehensive models of IPV. Applying the concept of structural interventions to IPV, we systematically reviewed the quantitative impact of such interventions for prevention of male-to-female IPV in LMIC in order to (a) highlight current opportunities for IPV research and programming and (b) demonstrate how structural interventions may provide an organizing framework through which to build an evidence base for IPV prevention.

**Methods:**

We identified articles by systematically searching PubMed and Web of Science, reviewing references of selected studies, and contacting 23 experts. Inclusion criteria included original research, written in English, published between January 2000 and May 2015 in the peer-reviewed literature. Studies evaluated the quantitative impact of structural interventions for the prevention of male-to-female IPV in LMIC through (a) IPV incidence or prevalence or (b) secondary outcomes theoretically linked to IPV by study authors. After initial screening, we evaluated full text articles for inclusion and extracted data on study characteristics, outcomes, and risk of bias, using forms developed for the review.

**Results:**

Twenty articles (16 studies) from nine countries met inclusion criteria, representing 13 randomized control trials and seven additional studies, all of which reported results from economic, social, or combined economic and social interventions. Standardized at *p* < 0.05 or 95 % confidence intervals not including unity, 13 studies demonstrated statistically significant effects for at least one primary or secondary outcome, including decreased IPV and controlling behaviors; improved economic wellbeing; enhanced relationship quality, empowerment, or social capital; reduced acceptability of IPV; new help seeking behaviors; and more equitable gender norms. Risk of bias, however, varied in meaningful ways.

**Conclusions:**

Our findings support the potential effectiveness of structural interventions for IPV prevention. Structural interventions, as an organizing framework, may advance IPV prevention by consolidating available evidence; highlighting opportunities to assess a broader range of interventions, including politico-legal and physical approaches; and emphasizing opportunities to improve evaluation of such interventions.

**Electronic supplementary material:**

The online version of this article (doi:10.1186/s12889-015-2460-4) contains supplementary material, which is available to authorized users.

## Background

Intimate partner violence (IPV) is the most common form of violence against women, with an estimated one in three women worldwide experiencing physical or sexual IPV during her lifetime [1]. Associated with a range of adverse physical, mental, and reproductive health consequences and intergenerational effects [1], researchers have granted increased attention to identifying determinants and effective intervention strategies over the past decade. Available evidence now highlights how gender inequities create power imbalances between men and women, which perpetuate violence [2], and provides evidence for a range of interventions for women who experience IPV [3]. Evidence for interventions that address known determinants in order to *prevent* IPV, however, remains under-prioritized. This is particularly true in low- and middle-income countries (LMIC), despite growing attention to IPV prevention in many of these settings [3].

The study of IPV often is framed in relation to gender inequities, particularly in low- and middle-income countries where women may experience severely restricted social and economic opportunities relative to men. In many settings, women experience constrained political power, lower socioeconomic status, unequal access to education, fewer employment opportunities, and restrictive gender expectations that promote male control of women, male sexual entitlement, and female subservience [2, 4–6]. These circumstances generate and maintain IPV risk for women.

Current research emphasizes that risk and vulnerability arise from gender inequity and attendant disparities operating at multiple levels of the social ecology, including dyadic, community, societal, and structural levels. Beginning from the highest or most encompassing level of the social ecology, gender inequities are seen in structural disparities. Such disparities may be economic, politico-legal, physical, or social, such as laws restricting inheritance for women. These structural disparities produce community inequities, such as inequitable expectations of male and female behaviors, and dyadic inequities, such as relative wealth disparities between male and female partners, at lower levels of the social ecology. Moving further down, these community and dyadic inequities increase individual risk and vulnerability for IPV; women have limited autonomy and agency to negotiate equitable treatment in their relationships and their partners may use violence to maintain these inequities [7]. In a reciprocal manner, these individual, dyadic, and community experiences also reinforce and reproduce gender inequities at higher levels of the social ecology, including structural disparities. For example, wealth disparities between men and women within partnerships and economic subordination of women to men within communities frequently limit the agency and political power of women, impacting their ability to argue for gender-equitable public policies that help protect women from violence.

This socio-ecologic framework is beginning to shape the focus of IPV interventions in LMIC. As classified by Ellsberg and colleagues [3], first generation interventions included services to support IPV survivors, legislation to criminalize violence against women, and policies and training to increase judicial effectiveness and police practices. Second generation interventions began with instrumental approaches targeting individual risk factors of vulnerable women, including attempts to modify their knowledge, attitudes, and behaviors [3, 8, 9]. This generation now includes interventions aimed to transform societal and community gender norms in order to prevent IPV [5, 8, 10]. A growing number of interventions target underlying gender inequities at multiple socio-ecologic levels to decrease IPV victimization, such as community mobilization interventions to address inequitable expectations of male and female behaviors at the community level and microfinance interventions to address economic subordination of women to men within households at the dyadic level.

Despite this promising growth, evaluation evidence for IPV prevention in LMIC remains limited. One prominent review recently synthesized available evidence for prevention of varied forms of violence against women [3]. The authors classified interventions by strategy, such as economic and livelihood interventions and response interventions that assist female victims of violence, ultimately describing four approaches with promising evidence: (1) participatory or community-driven development, (2) empowerment training to improve the agency of women, (3) workshops to address gender and behavioral norms among men and women, and (4) economic empowerment or income supplements combined with gender equality training [3]. This synthesis suggested important characteristics of successful approaches, including engaging numerous stakeholders in multiple ways, addressing socio-ecologic risk factors and inequitable gender norms, and supporting persons to develop nonviolent behaviors; however, it also highlighted the overall paucity of available evidence and need to expand the evidence base for prevention interventions, including assessing a broader range of interventions [3].

The concept of structural interventions may help to expand the current evidence base and broaden the range of evaluated interventions by providing a theory-driven framework through which to organize and critique available evidence. By definition, structural interventions aim to change structural factors, which are aspects of the economic, politico-legal, physical, and social environment that produce and reproduce risk [11]. Consistent with a socio-ecologic understanding of IPV risk (Table 1), such interventions modify systems, structures, and processes at the highest level of the social ecology in order to affect risk at multiple levels [11]. For example, effective structural interventions include legal regulations that restrict access to pesticides and mandate single-use packaging where pesticide poisoning is a common method of suicide [12], political policies that support syringe exchange and provision where injection drug use is a common method of HIV transmission [7], and participatory or community-driven development to transform inequitable gender norms where these norms increase IPV risk for women [3]. In opposition to behavioral approaches that encourage individual change, these approaches pattern individual behaviors, experiences, and risk by modifying aspects of the legal, political, or social environment that shape risk [11], including dyadic experiences and individual agency.

**Table 1:** Categories and risk factors for structural interventions for intimate partner violence

Category	Risk factor	Potential structural interventions
Economic	Poverty [47]	Microfinance programs for women [37]
	Limited economic opportunity [48]	
	Interpersonal (dyadic) economic inequality [49]: male control of family finances or wealth, women's economic dependence, and male unemployment with female employment [47]	Unconditional and conditional cash transfer programs [29]
Physical	Isolation of women to private spaces and limited public roles for women [49]	Community meeting spaces for women and girls [50]
		Limitations on alcohol outlet density [51–53]
	Alcohol outlet density [51–53]	
Politico-Legal	Legislation and practices that reinforce female subordination and discrimination (e.g., dowry, child marriage, restricted property rights) [54–57]	Legislation to facilitate women’s access to divorce [49]
		Legislation to protect survivors and prosecute perpetrators [49]
		Training for and monitoring of criminal justice and legal professionals on IPV-related policies and legislation [49]
	Limited sensitivity and awareness among service providers, law enforcement, and judicial actors [49, 58, 59]	
	Limited legal support for women and survivors of violence [49]	
Social	Social isolation [47] and limited freedom of movement	Social empowerment through community activities [49]
	Low educational level [60] of women [49]	
	Gender norms supporting male dominance [49]: existence or transgression of rigid gender roles [47, 49]	Educational entertainment media [41, 49]
	Community acceptance of interpersonal violence [47, 62]	Transformation of gender norms among men [61]

As an organizing framework, structural interventions historically guided a shift in global HIV programming from individual risk-based prevention (aiming to modify the behaviors of persons vulnerable to infection) to community and system-wide prevention (aiming to modify the contextual experiences that pattern individual risk) [7]. Whereas a similar shift from individual risk-based prevention (aiming to modify the behaviors of women vulnerable to IPV) to community and system-wide prevention (aiming to transform societal and community gender norms to prevent IPV) is beginning in second generation IPV interventions, the concept of structural interventions has been applied to IPV only intermittently. No known studies have utilized this framework to systematically evaluate evidence or explored its ability to guide a theory-driven shift for IPV prevention to date.

### Objectives

This systematic review aims to synthesize peer-reviewed evidence on the quantitative impact of structural interventions to prevent male-perpetrated IPV against women in LMIC. Although women experience myriad forms of violence, IPV is among the most common forms of violence against women and occurs in epidemic proportions [13]. Evaluation of evidence for IPV interventions has been under-prioritized in LMIC relative to high-income countries [14], despite 24–71 % of women reporting lifetime physical or sexual IPV in LMIC [13]. Although IPV also affects women in high-income countries, structural interventions may be particularly impactful in LMIC due to the relative significance of gender inequality in shaping risk and the potential for structural interventions to impact gender inequities at all levels of the social ecology. By describing the range of evaluated interventions and considering evidence of their effectiveness, this study (a) highlights current opportunities for IPV research and programming in these settings and (b) demonstrates how structural interventions may provide an organizing framework through which to assess and build an evidence base for IPV prevention interventions in LMIC.

## Methods

### Eligibility criteria

We conducted a per-protocol review following PRISMA guidelines for systematic reviews [15] (review protocol unregistered, see Additional file 1: Table S1 for PRISMA checklist). We systematically searched PubMed and Web of Science for articles that evaluated structural interventions for IPV. Studies eligible for inclusion were (1) original research, (2) written in English, and (3) published between January 1, 2000 and May 23, 2015 in the peer-reviewed literature. Such studies (4) evaluated the impact of a structural intervention for primary and secondary prevention of male-to-female IPV in (5) World Bank-defined LMIC through (6) quantitative evaluation of the impact on (a) IPV incidence or prevalence or (b) secondary outcomes theoretically linked to IPV incidence or prevalence by the authors. Secondary outcomes included intermediate outcomes hypothesized to explain the impact of the intervention on IPV, such as increased financial autonomy and security among women or decreased acceptability of spousal violence among men.

We defined structural interventions as interventions that address economic, physical, politico-legal, or social environments that produce or reproduce IPV risk, in contrast to individual interventions that target individual knowledge, attitudes, and behaviors [11]. We included structural approaches that target structural risk at local levels to permit inclusion of rigorous study designs [7]: for example, randomized control trials (RCT) of microfinance delivered to individual women to understand the potential impact of ameliorating poverty-related stressors, diminishing intra-household gender inequalities, and affecting community norms at scale. Consistent with the definition of structural interventions [11], however, we excluded interventions that targeted risk behaviors without modifying aspects of the economic, physical, politico-legal, or social environments that produce and reproduce risk for IPV. These included processes within healthcare systems, such as one-stop centers for survivors of gender-based violence [3], and advocacy interventions aimed to empower and link IPV survivors to community services [16].

We defined primary and secondary prevention interventions as those aimed to prevent or reduce IPV, without consideration of prior exposure to violence [3]. Whereas primary prevention aims to prevent initial IPV and secondary prevention aims to prevent ongoing IPV, current epidemiologic measures of IPV have limited sensitivity to measure IPV patterns, and structural interventions address aspects of the environment that likely promote initial and sustained violence. We did not include tertiary prevention strategies, which aim to prevent negative health or social sequelae among victims following IPV, in alignment with the goal to investigate interventions that reduce the incidence or prevalence of IPV.

We focused on peer-reviewed literature to capture the highest quality research, and limited publications to English due to constraints on translation and evaluation of non-English language publications. We did not specify population restrictions, given the disproportionate prevalence of IPV and significant structural challenges that subpopulations, such as adolescents, injection drug users, and sex workers, experience. Similarly, we did not limit study designs, outcomes, or statistical summary measures. There exists active debate about appropriate outcomes for IPV intervention studies (e.g., IPV, proximal measures of social change, or outcomes under the control of women) [17, 18]. Additionally, structural interventions may require diverse evaluation strategies; RCTs may be most appropriate when interventions alter single, proximate risk factors and other influences are known and measured [7]. Although understanding process outcomes is important [7], these issues were beyond the scope of this review. We privileged quantitative measures in order to compare impact across studies.

### Search strategy

**Fig. 1:**
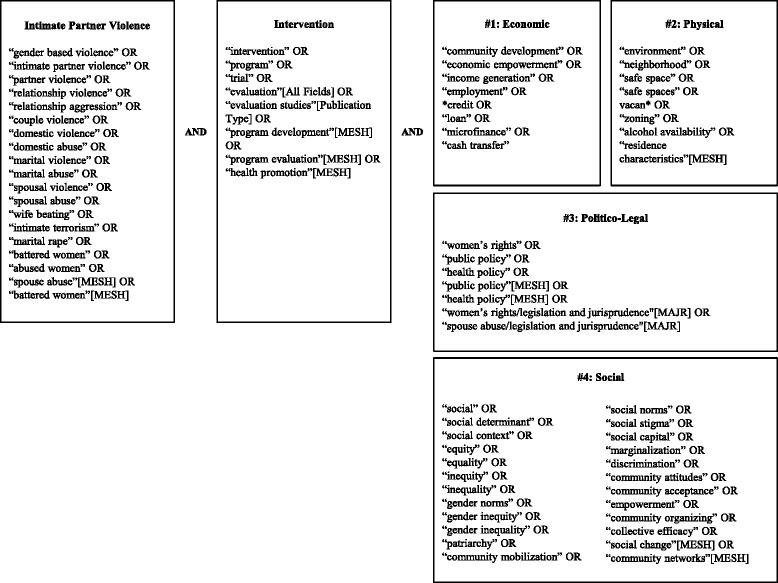
Search terms

**Fig. 2:**
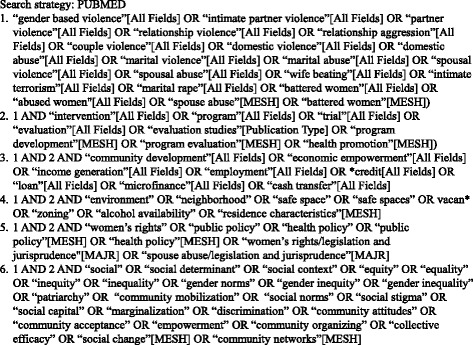
Electronic search strategy for PubMed

We performed four searches that combined three search themes (IPV, intervention, and one of four structural categories) with the Boolean operator “and” (Figs. 1 and 2) on March 14, 2013 (January 2000 to March 2013) and May 23, 2015 (March 2013 to May 2015). Each search theme included a comprehensive list of terms intended to account for historical and disciplinary heterogeneity in terminology. We selected search terms through a multistep process. We first identified potential terms through expert identification, published reviews, and key terms in relevant articles. We then evaluated and selected terms based on their sensitivity (ability to identify relevant literature) and uniqueness (ability to identify articles not captured by other search terms). Index terms identified in PubMed were applied in Web of Science as phrases bracketed by quotations.

Secondary search strategies included manually searching reference lists of articles identified for data extraction and contacting 23 experts to recommend additional studies. These experts included prominent researchers and first and senior authors for 12 articles identified for data extraction during the original submission, including two articles [19, 20] that were found ineligible during data extraction: one because the outcome definition did not ensure IPV measurement [19] and the other because statistical analyses of relevant outcomes were not published [20]. Among 23 experts contacted, seven responded. They recommended 26 articles cumulatively, one of which met inclusion criteria. Given time limitations, we did not repeat this step during manuscript revision. We also planned, but did not conduct, hand-searches of key journals identified from electronic searches and reference list reviews due to limited consensus on key journals from these steps.

### Study selection, data extraction, and analysis

One author (CB) screened all records from the database search, reference lists, and expert recommendations. Each author (CB, WW, EB, and RS) independently assessed a selection of full-text articles for eligibility using a form developed and piloted on five randomly selected articles. WW and EB extracted data independently using a form developed and piloted on three randomly selected articles. These authors extracted data in duplicate for the original submission; research assistants checked extracted data during the review update. We resolved discrepancies at each stage by consensus. In instances where multiple articles reported on the same study, we extracted data from each article separately due to varied theoretical frameworks, analytic samples, and outcome measures. Planned data extraction included intervention characteristics (intervention and comparison exposures, allocation or targeting, and exposure period) and study characteristics (data source, study design, sampling strategy, sample size, inclusion criteria, follow-up period, exposure assessment, coverage, and unintended harms). We recorded data on primary outcomes (IPV incidence or prevalence) and secondary outcomes (intermediate outcomes linked to IPV incidence or prevalence by study authors) to facilitate comparison across studies. Statistics included those from the most saturated models presented by authors, privileging intention to treat analyses. Risk of bias evaluation differed for RCTs and other study designs. Questions captured selection, performance, detection, analysis, and reporting biases and IPV-specific issues, reflecting PRISMA [21], Cochrane Collaboration [22], and WHO recommendations for IPV research [23]. This evaluation focused on study level risk of bias, with selective reporting within studies recorded and outcome level risk included in the discussion of intervention effects. We collected information on risk of bias as descriptive data; these did not influence data synthesis, as meta-analysis was not planned for statistical, methodological, and programmatic reasons [21]. The diversity of study designs, outcome measures and definitions, and contexts inhibited quantitative synthesis.

Planned data differed from collected data for risk of bias analyses. Heterogeneous study designs and data presentation styles limited the ability to calculate intervention coverage and attrition consistently. Calculations of intervention coverage alternately reflected the percentage of interviewed or enrolled participants among potential participants, all volunteers, or eligible volunteers or the percentage meeting varied participation thresholds among randomized or enrolled participants. Attrition similarly described loss to follow-up among randomized, interviewed, or participating individuals.

Although we did not contact authors for further information, we invited first authors of the 10 studies included in the original submission to comment on any aspect of the manuscript, including data presentation and interpretation. As only one author responded, without expressed concerns, we did not repeat this step during the review update.

## Results

**Fig. 3:**
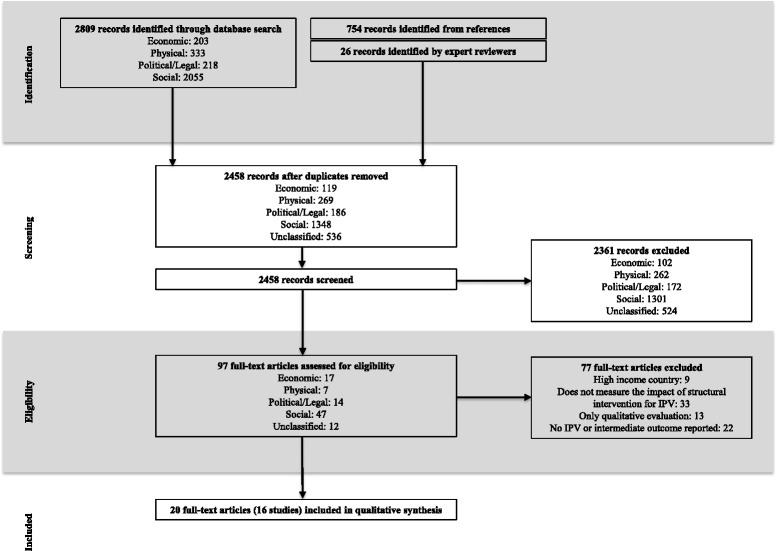
Study selection

In total, we identified 3589 records for screening, of which 2458 were unique (Fig. 3). Ninety-seven full-text articles were assessed for eligibility, yielding 22 articles (18 studies) that met inclusion criteria. A further two articles were eliminated during data extraction for the original submission, as previously described. Twenty articles, representing 16 studies, are included in this review (Additional file 2: Table S2) [6, 24–42].

### Study characteristics

The articles described interventions conducted between 1992 and 2012 in nine countries: Bangladesh [24], China [39], Côte d’Ivoire [28, 30], Ecuador [29], Ethiopia [40], India [26, 36], Mexico [25], South Africa [31–34, 37, 38, 41], and Uganda [6, 27, 35, 42] (Additional file 2: Table S2). Eleven of 20 articles used rural samples [24, 25, 27, 28, 30, 31, 33, 34, 37, 38, 42], by intervention design or analytic restriction. Eight articles, representing seven studies, engaged men and women [6, 27, 28, 31, 32, 35, 41, 42]; seven articles (four studies) targeted women exclusively to address outcomes of interest [24, 25, 29, 33, 34, 37, 38]; and five articles (five studies) involved men exclusively [26, 30, 36, 39, 40]. Common restrictions included socio-demographic vulnerabilities, such as women or households with low socioeconomic status [24, 25, 27, 29, 33, 34, 37, 38] and communities with high marginality [25], informal land tenure [32], or increased exposure to armed conflict [27]. Twelve articles (11 studies) restricted interventions or analytic samples to reproductive-aged or young persons [6, 24, 25, 27, 29, 31, 32, 35, 36, 39, 40, 42]. The mean sample size across studies was 1061.5 (IQR 489.5–1274.3) for outcome models or study samples, where authors did not report sample sizes for outcome models.

### Structural interventions

All articles described social, economic, or social and economic interventions. Economic interventions examined the utility of microfinance and cash transfers, including BRAC-led microfinance and skills programs in rural Bangladesh [24], cash transfers and microenterprise training in post-conflict northern Uganda (Women’s Income Generating Support, WINGS) [27], the national conditional cash transfer program in Mexico (*Oportunidades*) [25], and the national unconditional cash transfer program in Ecuador (*Bono de Desarrallo Humano*, BDH) [29].

Social interventions tested a variety of participatory learning, community mobilization, and multimedia approaches. Participatory learning programs aimed to improve sexual health among young men and women in South Africa (Stepping Stones) [31], enhance gender equitable attitudes among young men in China [39], and promote bystander behaviors among adolescent male cricket athletes in India (*Parivartan*) [36]. Community mobilization interventions focused on critical analysis and discussion of power inequities among men and women in urban Uganda (SASA!) [6, 35] and IPV-related norms and behaviors in rural Uganda (Safe Homes and Respect for Everyone, SHARE) [42] and included a compilation of workshops and campaigns on gender relations and violence against women in North India, led by and targeted to men (Men’s Action to Stop Violence Against Women, MASVAW) [26]. Interventions in rural Côte d’Ivoire [30] and urban Ethiopia (Male Norms Initiative) [40] combined community-based programming with discussion groups for men. A media campaign promoted implementation of domestic violence legislation and social norm change related to domestic violence in South Africa (Soul City Fourth Series, SC4) [41].

Four studies, reported in seven articles, combined economic and social approaches: (1) microfinance and gender training in South Africa (Intervention with Microfinance for AIDS and Gender Equity, IMAGE) [33, 34, 37, 38]; (2) livelihoods training (Creating Futures) and sexual health training (Stepping Stones) in South Africa [32]; (3) cash transfers, microenterprise training, and gender and couples training in post-conflict northern Uganda (Women Plus) [27]; and (4) group savings for women with gender dialogue groups for couples (or women and their male family members) in rural Côte d’Ivoire [28].

Thirteen articles discussed prospective RCTs, with a mean follow up period of 24.3 months from study initiation. Three articles used longitudinal designs: a non-randomized trial, in which researchers allocated urban middle schools to intervention and wait list control conditions purposively to reduce potential contamination due to geographical proximity [36]; an interrupted time series evaluation, which measured outcomes successively among intervention participants [32]; and a before-after analysis, in which researchers collected data from a unique stratified random sample at baseline and follow-up and defined intervention and comparison groups retrospectively [41]. Additionally, three articles reported on cross-sectional studies, two of which used study-specific surveys with non-random or unspecified sampling [24, 26] and one of which used a nationally representative sample [25]. Exposure periods in non-RCTs ranged from a measured seven months [41] to a potential maximum of seven years [24].

All articles, except one [32], reported comparisons with unexposed or standard-of-care comparators. Although this article reported a proof of concept study, rather than an impact evaluation [32], we included it because it represents a unique contribution to the literature. The study evaluated the combination of Stepping Stones, a widely implemented social intervention, and Creating Futures, an economic intervention providing livelihoods training *without* supplementing participant income. Two articles, by contrast, included multiple comparators. Kim et al. [34] reported a three-way comparison to disentangle IMAGE intervention components, comparing combined microfinance and social components, microfinance only, and no exposure. Green et al. [27] reported two trials: first comparing cash transfers and microenterprise training to waitlist control and then comparing cash transfers and microenterprise training to its combination with gender and couples training.

### Intervention outcomes

Outcome measures varied across studies (Tables 2 and 3, Additional file 3: Table S3). Sixteen articles reported IPV as an outcome, including single types of IPV (i.e., physical IPV [6, 25, 28–30, 32, 39, 42], psychological IPV [25, 29, 39, 42], sexual IPV [6, 25, 28, 30, 32, 35, 42], economic IPV [28], and controlling behaviors [27, 29, 33, 34, 37]) and aggregate measures (i.e., physical or psychological IPV [24, 27, 39], physical or sexual IPV [25, 28, 30–34, 37, 40], and physical, sexual, or psychological IPV [40]). Authors described using behavioral measures of IPV in 15 articles [6, 24, 25, 27–30, 32–35, 37, 39, 40, 42], although several reported behaviors incompletely [24, 25, 34] and few discussed validation of selected questions in the study context. Nearly all studies measured recent IPV: differentially defined as three [32, 39], four [24], six [40], eight [27], or 12 months [6, 25, 28, 30, 31, 33–35, 37, 42]. Only one study measured lifetime physical IPV or did not specify a recall period [29]. In many articles, self-reported experience of IPV was measured among married or partnered women [6, 24, 25, 28–30, 34, 35, 37], by sampling or analytic restriction. By contrast, several articles summarized male-reported perpetration of violence [31, 32, 39, 40, 42].

**Table 2:** Study effects for IPV outcomes

First author (year)	Physical	Psychological^1^	Sexual	Economic	Controlling behaviors	Multiple types
Abramsky (2014) [6]	NS		NS			
Ahmed (2005) [24]						NS
Bobonis (2013) [25]	NS	NS	NS			NS
Das (2012) [26]						
Green (2015) [27] Trial 1					*	NS
Trial 2					NS	NS
Gupta (2013) [28]	NS		NS	*		NS
Hidrobo (2013) [29]	NS	NS			*	
Hossain (2014) [30]	NS		NS			NS
Jewkes (2008) [31]						NS
Jewkes (2014) [32]	NS		* / NS^2^			* / NS^2^
Kim (2007) [33]					NS	*
Kim (2009) [34]					NS	* / NS^3^
Kyegombe (2014) [35]			NS			
Miller (2014) [36]						
Pronyk (2006) [37]					NS	*
Pronyk (2008) [38]						
Pulerwitz (2015) [39]	NS	* / NS^4^				*
Pulerwitz (2015) [40]						NS / unknown^5^
Usdin (2005) [41]						
Wagman (2015) [42]	* / NS^6^	* / NS^7^	* / NS^6^			

*Significant at *p* < 0.05 or 95 % confidence interval not including unity

*NS* = not significant

^1^Psychological IPV includes author-defined psychological, emotional, and verbal violence and threats of physical violence

^2^Significant for women*, not significant for men

^3^Significant for IMAGE v. control*, not significant for microfinance v. control or IMAGE v. microfinance

^4^Significant for workers*, not significant for students

^5^ *P*-values not presented for all statistics from multivariate regression models

^6^Significant for women at 35 months*, not significant for men at 35 months, women at 16 months, or men at 16 months

^7^Significant for men at 16 months*, not significant for women at 35 months, men at 35 months, or women at 16 months

**Table 3:** Study effects for economic and social outcomes

First author (year)	Economic	Social			
	Economic wellbeing	Help seeking or receipt	Attitudes toward IPV	Gender norms	Other social pathways^1^
Abramsky (2014) [6]		NS^2^			
Ahmed (2005) [24]					
Bobonis (2013) [25]					
Das (2012) [26]				* / NS^3^	
Green (2015) [27] Trial 1	*			NS	* / NS^4^
Trial 2	NS			NS	* / NS^5^
Gupta (2013) [28]			*	NS	
Hidrobo (2013) [29]					
Hossain (2014) [30]			NS		*
Jewkes (2008) [31]					
Jewkes (2014) [32]	* / NS^6^			*	* / NS^7^
Kim (2007) [33]	* / NS^8^		NS		* / NS^9^
Kim (2009) [34]	* / NS^10^		* / NS^11^		* / NS^12^
Kyegombe (2014) [35]					* / NS^13,14^
Miller (2014) [36]			NS	*	
Pronyk (2006) [37]	* / NS^15^		NS		* / NS^16^
Pronyk (2008) [38]					NS
Pulerwitz (2015) [39]				*	
Pulerwitz (2015) [40]				* / NS^17^	
Usdin (2005) [41]		*	* / NS^18^		*
Wagman (2015) [42]					

*Significant at *p* < 0.05 or 95 % confidence interval not including unity

*NS* not significant

^1^Other social pathways include a range of author-defined outcomes, including indicators for relationship quality, empowerment, social capital, and collective action

^2^Limited to appropriate community response to women experiencing IPV in past year, as indicators measuring acceptability of physical violence by a man against his partner and acceptability of a woman refusing sex changed from baseline to follow-up

^3^Measured as 8 scales for activist v. control (women’s role/autonomy*, gender roles*, domestic work*, masculinity*, sexuality*, knowledge of women/child laws*, women do “traditional women’s work”*, men do “traditional male work”*) and influenced v. control (women’s role/autonomy*, gender roles*, domestic work*, masculinity*, sexuality*, knowledge of women/child laws, women do “traditional women’s work”*, men do “traditional male work”*)

^4^Measured as 2 indicators for men and women (self-reported autonomy/influence in purchases, partner relationship index*) and women only (self-reported autonomy/influence in purchases, partner relationship index*)

^5^Measured as 5 indicators for women only (self-reported autonomy/influence in purchases, partner relationship index*, partner support index overall*, partner support index: family*, partner support index: business)

^6^Measured as 12 indicators for women (mean earnings last month*, currently studying, frequency of livelihood strengthening efforts, work stress, feelings about work situation mean score*, financially supporting kids*, receiving a grant*, hungry every day or week, borrowing food or money weekly or more often, stole in last month due to lack of food or money*, crime participation score, very difficult to find 200 rand in an emergency*) and men (mean earnings last month*, currently studying, frequency of livelihood strengthening efforts*, work stress*, feelings about work situation mean score*, financially supporting kids, receiving a grant, hungry every day or week, borrowing food or money weekly or more often, stole in last month due to lack of food or money*, crime participation score, very difficult to find 200 rand in an emergency*)

^7^Measured as 4 indicators for women (relationship control scale, any club or group involvement*, active in church, community cohesion score) and men (relationship control scale*, any club or group involvement, active in church, community cohesion score)

^8^Measured as 3 indicators (estimated household asset value >2000 rand*, expenditure on shoes and clothing >200 rand/year, savings group membership)

^9^Measured as 9 indicators (more self-confidence, greater financial confidence, challenging gender norms, autonomy in decision-making, perceived contribution to household valued by partner, household communication regarding sexual matters in the past year*, supportive partner relationship, greater social group membership, takes part in collective action)

^10^Measured as 9 indicators for microfinance v. control (greater food security, household asset value > US$300*, greater expenditure on home improvements, better able to pay back debt*, membership in savings group*, able to meet basic needs in past year*, possesses bank account, better perception of household economic well-being, has not had to beg in past month*), IMAGE v. control (greater food security, household asset value > US$300, greater expenditure on home improvements*, better able to pay back debt, membership in savings group, able to meet basic needs in past year, possesses bank account, better perception of household economic well-being, has not had to beg in past month), and IMAGE v. microfinance (greater food security, household asset value > US$300, greater expenditure on home improvements, better able to pay back debt, membership in savings group, able to meet basic needs in past year, possesses bank account, better perception of household economic well-being, has not had to beg in past month)

^11^Significant for IMAGE v. microfinance*, not significant for microfinance v. control or IMAGE v. control

^12^Measured as 9 indicators for microfinance v. control (greater self-confidence*, greater financial confidence, challenges gender norms, supportive partner relationship, autonomy in decision-making, perceived contribution to household, larger social network, greater sense of community support, greater solidarity in crisis), IMAGE v. control (greater self-confidence, greater financial confidence, challenges gender norms, supportive partner relationship, autonomy in decision-making, perceived contribution to household*, larger social network, greater sense of community support, greater solidarity in crisis), and IMAGE v. microfinance (greater self-confidence*, greater financial confidence, challenges gender norms, supportive partner relationship*, autonomy in decision-making, perceived contribution to household*, larger social network, greater sense of community support, greater solidarity in crisis*)

^13^Measured as 11 indicators for women (feels able to refuse sex with partner, made important decisions jointly with partner all/most of the time*, male partner helps with housework, male partner helps look after children, shown appreciation many times for work partner does in the house, shown appreciation many times for work partner does outside the house, discussed number of children you would like to have, openly asked what partner likes during sex, openly told partner what you like during sex, discussed things that happen to both you and partner during the day, discussed your worries/feelings) and 10 indicators for men (made important decisions jointly with partner all/most of the time*, male partner helps with housework*, male partner helps look after children*, shown appreciation many times for work partner does in the house*, shown appreciation many times for work partner does outside the house*, discussed number of children you would like to have*, openly asked what partner likes during sex*, openly told partner what you like during sex*, discussed things that happen to both you and partner during the day, discussed your worries/feelings*)

^14^The authors indicate that “question wording/item construction changed between baseline and follow-up to improve face validity” (p. 6), yet it is unclear which indicators changed from the information reported [35]. All potentially relevant measures are included

^15^Measured as 5 indicators (estimated value of selected household assets >2000 rand*, membership in savings group, greater food security, per person expenditure on clothing or shoes >200 rand, children aged 10–19 years attending school)

^16^Measured as 9 indicators (more participation in social groups, taken part in collective action, greater perception of community support in a time of crisis, belief that the community would work together toward common goals, more positive attitude to communal ownership, more self-confidence, greater challenge of established gender roles, communication with intimate partner about sexual matters in past 12 months, communication with household members about sexual matters in past 12 months*)

^17^Significant for GE + CE v. CE and control*, not significant for CE v. control

^18^Measured as difference between baseline and follow-up for 10 indicators defined as “personal attitudes” and “subjective norms” (I agree that domestic violence is a serious problem*, I disagree that violence between a man and a woman is a private affair*, I agree that no woman ever deserves to be beaten*, I disagree that women who are abused are expected to put up with it*, I disagree that in my culture it is acceptable for a man to beat his wife, I disagree, as head of the household, a man has the right to beat a woman, my community agrees that domestic violence is a serious problem*, my community disagrees that violence between a man and a woman is a private affair*, my family agrees that no woman ever deserves to be beaten*, my family disagrees that women who are abused are expected to put up with it*) and by level of media exposure at follow-up (I agree that domestic violence is a serious problem*, I disagree that violence between a man and a woman is a private affair*, I agree that no women ever deserves to be beaten*, I disagree that women who are abused are expected to put up with it*, I disagree that in my culture it is acceptable for a man to beat his wife*, I disagree, as head of the household, a man has the right to beat a woman*, my community agrees that domestic violence is a serious problem, my community disagrees that violence between a man and a woman is a private affair*, my family agrees that no woman ever deserves to be beaten*, my family disagrees that women who are abused are expected to put up with it*)

Based on the most saturated models, privileging intention to treat analyses, and standardizing statistical significance at *p* < 0.05 or 95 % confidence intervals not including unity, nine of 16 articles reported a statistically significant difference in the desired direction for at least one IPV indicator. Among economic interventions, WINGS, which included cash transfers and microenterprise training in post-conflict Uganda, was associated with decreased odds of controlling behaviors compared to waitlist control [27]. BDH, which analyzed the national unconditional cash transfer program in Ecuador, was associated with decreased odds of controlling behaviors in the full sample [29]. Among social interventions, a participatory gender learning program in China was associated with decreased psychological and physical or psychological IPV perpetration among workers and students at follow-up [39]. SHARE, a community mobilization and HIV intervention, also noted decreased female-reported physical and sexual IPV experience at 35 months and male-reported psychological IPV perpetration at 16 months [42]. Combined economic and social interventions included group savings for women and gender dialogue groups for couples in rural Côte d’Ivoire [28]; these were associated with decreased odds of economic IPV compared to group savings only. The combination of Stepping Stones, a participatory learning program, and Creating Futures, a livelihoods intervention, was associated with decreasing sexual IPV and physical or sexual IPV among female participants over time [32], and IMAGE, a microfinance and participatory learning program, was associated with decreased risk of physical or sexual IPV [33, 34, 37].

By contrast, SASA! in Uganda [6, 35], BRAC in Bangladesh [24], *Oportunidades* in Mexico [25], Stepping Stones in South Africa [31], the Male Norms Initiative in Ethiopia [40], and the addition of male discussion groups to community-based prevention in Côte d’Ivoire [30] did not report significant associations at the *p* < 0.05 level for any IPV indicators included in this review. Interventions with multiple comparators also showed non-significant effects for specific intervention components. Cash transfers and microenterprise training provided in WINGS were associated with reduced IPV, yet the addition of gender and couples training to this (Women Plus) was not associated with further IPV reduction [27]. Conversely, IMAGE was associated with reduced risk of past-year physical or sexual IPV compared to waitlist control [33, 34, 37], but a disentanglement study showed no significant effect for the intervention when IMAGE was compared to microfinance alone [34].

Other outcome measures included varied indicators of economic wellbeing [27, 32–34, 37], IPV-related help seeking or receipt [6, 41], attitudes toward IPV [28, 30, 33, 34, 36, 37, 41], gender norms [26–28, 32, 36, 39, 40], and other social pathways related to women’s equity, autonomy, or agency at varied levels of the social ecology, including improved relationship quality, personal empowerment, greater social capital, and collective action [27, 30, 32–35, 37, 38, 41]. Economic interventions with positive effects included WINGS, which was associated with improved economic wellbeing and relationship quality [27].

Among social interventions, SASA! was associated with improvement in nine of 10 indicators measuring relationship quality among men [35], although only one of 11 indicators improved among women [35] and the prevalence of appropriate community responses to women experiencing IPV did not improve significantly [6]. In MASVAW, a community action program targeted toward men in North India, activist men (active intervention members) and influenced men (non-activists in intervention communities) demonstrated statistically greater agreement with gender equitable norms compared to controls for all scales, except knowledge of women and child laws among influenced men [26]. In Côte d’Ivoire, men assigned to discussion groups and community-based prevention programming reported improved ability to control hostility and manage conflict and greater participation in gendered household tasks than men assigned to community-based prevention programming only [30]. The between-group comparison on change score similarly differed for gender attitudes among adolescent males in Parivartan, a bystander intervention in India [36]. The Gender Equitable Men scale revealed more equitable attitudes among male workers and students assigned to participatory health programming in China [39] and group education and community engagement in Ethiopia [40]. Further, increased Soul City media exposure was associated with higher prevalence of collective action and help seeking behaviors, and eight of ten indicators suggested reduced acceptability of IPV at follow-up than baseline [41].

Combined economic and social interventions included Women Plus in Uganda, which was associated with improved relationship quality, general partner support, and partner support of household activities among women assigned to receive the economic (cash transfers and microenterprise training) and social intervention (gender and couples training) versus the economic intervention only [27]. The combination of group savings for women with gender dialogue groups for couples in rural Côte d’Ivoire demonstrated greater improvement in attitudes condoning spousal abuse than group savings alone [28]. Similarly, the combination of Stepping Stones, a participatory learning program, and Creating Futures, a livelihoods intervention, was associated with improvement in gender norms reported by men and women, relationship equity reported by men, and club or group involvement reported by women as well as half of indicators measuring economic wellbeing [32]. IMAGE was associated with increased economic wellbeing [33, 34, 37] and social capital or empowerment [33, 34, 37] compared to unexposed controls, in addition to less endorsement of attitudes condoning IPV compared to microfinance only participants [34]. Only two articles did not report statistically significant associations for any economic or social indicators included in this review [6, 38]. These articles, however, reported on SASA! in Uganda and IMAGE in South Africa; both interventions were associated with positive effects in other articles [33–35, 37].

Five studies noted unintended harms. Study authors discussed that passive and active BRAC members (receiving savings only and savings and credit, respectively) reported increased odds of IPV compared to skilled members (receiving savings, credit, and training). They interpreted this as time-bound risk, which dissipates with longer participation, as women exposed to more intervention components also had participated longer in the intervention [24]. *Oportunidades* was associated with increased threats of violence [25]; however, this change was not statistically significant. In Uganda, the introduction of gender and couples training to cash transfers and microenterprise training improved couples’ relationship quality *without* increasing endorsement of gender equitable norms, financial autonomy, or economic success among women or reducing IPV prevalence [27]. The authors interpreted these findings positively, suggesting the couples-focused intervention may have initiated a process of social learning, beginning with improved relationship quality, that might result in increased financial autonomy and decreased use of violence [27]. They also noted, however, that men may have learned new ways to establish control in marital relationships, “[influencing] their female partners … by spending time with them, talking to them, and persuading them to do what they want” (p. 187) [27]. BDH demonstrated disparate intervention effects where absolute and relative inequities intersected; women with less than six years of schooling and education levels equal to or more than their partners had greater odds of experiencing IPV at follow-up at the *p* < 0.1 level in stratified analyses [29]. Additionally, female participants exposed to the combination of Stepping Stones and Creating Futures reported increased heavy drinking over time, which the authors linked theoretically to their rising incomes [32].

### Risk of bias

Risk of bias assessment revealed limitations affecting the quality and generalizability of findings (Tables 4 and 5). Reflecting limitations in study design or reporting bias, four of 13 articles describing RCTs [6, 27, 28, 42] reported calculating and enrolling a sufficiently large sample to support statistical inference for IPV outcomes. Only one of these 13 articles described allocation concealment [6], and no authors clearly identified blinding of the outcome assessment. All primary outcome analyses (Additional file 3: Table S3) controlled statistically for potential confounders [6, 27, 29–31, 33–35, 37, 38, 40, 42] or confirmed successful randomization by analyzing selected indictors at baseline and follow-up [28]. Nine of 13 articles presented intention to treat analyses; clear descriptions of this approach were missing from articles reporting IMAGE [33, 34, 37] and the Male Norms Initiative in Ethiopia [40]. Although all studies, except two [35, 40], appeared to report each outcome described in the methods among study results, only five articles clearly stated that implementation and analyses proceeded independently of funders [28, 33, 34, 37, 42].

**Table 4:** Risk of bias in randomized control trials

	Abramsky (2014) [6]	Green (2015) [27]	Gupta (2013) [28]	Hidrobo (2013) [29]	Hossain (2014) [30]	Jewkes (2008) [31]	Kim (2007) [33]	Kim (2009) [34]	Kyegombe (2014) [35]	Pronyk (2006) [37]
Study design										
Prospective identification of intervention and comparison groups	Yes	Yes	Yes	Yes	Yes	Yes	Yes	Partial^1^	Yes	Yes
Baseline and follow-up measurement of intervention and comparison groups	Yes	Yes	Yes	Yes	Yes	Yes	Yes	Partial	Yes	Yes
Selection bias										
Sample size calculation	Yes	Yes	Yes	NR	NR	Yes^2^	Yes^3^	NR	NR	Yes^3^
Random sequence generation^4^	Yes	Yes + ^5^	Yes	Yes	Yes	Yes+	Yes	Yes	Yes	Yes
Allocation concealment	Yes^6^	No	No	NR	NR	No	NR	NR	NR	NR
Blinding of outcome assessment	NR	NR	No	NR	No	NR	NR	NR	NR	NR
Detection bias										
Consistent outcome measurement across intervention and comparison groups	Yes	Yes	Yes	Yes	Yes	Yes	Yes	Yes	Yes	Yes
Analysis										
Statistical control for confounding	Yes	Yes	No^7^	Yes	Yes	Yes	Yes	Yes	Yes	Yes
Intention to treat analysis	Yes	Yes	Yes	Yes	Yes	Yes	NR	NR	Yes	NR
Reporting bias										
Complete reporting of outcomes described in methods in results	Yes	Yes	Yes	Yes	Yes	Yes	Yes	Yes	No	Yes
Reporting bias: conflicts of interest										
Implementation and analysis independent from funders	NR	NR	Yes	NR	NR	NR	Yes	Yes	NR	Yes
Reporting bias: adherence to recommendations for IPV research										
Age ≥15 for IPV questions	Yes	No	Yes	NR	Yes	Yes	Yes	Yes	NR	NR
IPV-specific training for interviewers	Yes	Yes	Yes	NR	Yes	NR	Yes	Yes	NR	Yes
IPV referral information or protocols	Yes	NR	Yes	NR	Yes	NR	Yes	NR	Yes	NR

*NR* not reported

^1^The authors did not report baseline measurement for the microfinance only intervention

^2^A sample size calculation was performed for HIV incidence, not IPV

^3^A sample size calculation was performed, but investigators were not able to enroll a sufficient number of clusters to adhere to minimum sample size calculations

^4^ *NR* not reported, Yes = randomization reported, and Yes + = randomization and randomly generated allocation sequence reported

^5^A public lottery was used for WINGS v. control, and a randomization algorithm was used for W+ v. WINGS

^6^Interviewers were blinded to allocation at baseline, not follow-up

^7^The authors report that no covariates were included in intention to treat analyses because randomization was successful

**Table 5:** Risk of bias in randomized control trials and other study designs

	Randomized control trial			Other study designs						
	Pronyk (2008) [38]	Pulerwitz (2015) [40]	Wagman (2015) [42]	Ahmed (2005) [24]	Bobonis (2013) [25]	Das (2012) [26]	Jewkes (2014) [32]	Miller (2014) [36]	Pulerwitz (2015) [39]	Usdin (2005) [41]
Study design										
Prospective identification of intervention and comparison groups	Yes	Yes	Yes	No	No	No	Intervention only	Yes	Intervention only	No
Baseline and follow-up measurement of intervention and comparison groups	Yes	Yes	Yes	No	No	No	Intervention only	Yes	Intervention only	Yes
Selection bias										
Sample size calculation	NR	NR	Yes	NR	NR	No	NR	NR	NR	NR
Random sequence generation^1^	Yes	Yes	Yes+	---	---	---	---	---	---	
Allocation concealment	NR	No	No	---	---	---	---	---	---	---
Blinding of outcome assessment	NR	NR	NR	---	---	---	---	---	---	---
Equivalent eligibility criteria in intervention and comparison groups	---	---	---	Unclear^2^	Yes	Yes	Intervention only	Yes	Intervention only	Yes
Detection bias										
Consistent outcome measurement across intervention and comparison groups	Yes	Yes	Yes	Yes	Yes	Yes	Intervention only	Yes	Yes	Yes
Analysis										
Statistical control for confounding	Yes	Yes	Yes	Yes	Yes	Yes	No	Yes	Partial^3^	Yes
Intention to treat analysis	Yes	NR	Yes	---	---	---	---	---	---	---
Reporting bias										
Complete reporting of outcomes described in methods in results	Yes	Partial^4^	Yes	Yes	Yes	Yes	Yes	Yes	Partial	Unclear
Reporting bias: conflicts of interest										
Implementation and analysis independent from funders	NR	NR	Yes	NR	NR	NR	NR	NR	NR	Partial^5^
Reporting bias: adherence to recommendations for IPV research										
Age ≥15 for IPV questions	N/A	Yes	Yes	Yes	Yes	N/A	Yes	N/A	Yes	N/A
IPV-specific training for interviewers	N/A	NR	Yes	Unclear	NR	N/A	NR	N/A	NR	N/A
IPV referral information or protocols	N/A	NR	Yes	NR	NR	N/A	NR	N/A	NR	N/A

*NR* not reported

*N*/*A* not applicable

^1^ *NR* not reported, Yes = randomization reported, and Yes + = randomization and randomly generated allocation sequence reported

^2^It is unclear whether equivalent criteria were used to define “low-income” in intervention and comparison households based on reported methods

^3^It appears that authors used chi square tests or bivariate regression for IPV outcomes, and multivariate regression for analyses of gender norms

^4^The authors report all outcomes described in the methods among the results; however, they do not show full, adjusted effect sizes for all outcomes

^5^Evaluation reported to be conducted and managed by independent researchers

Observational studies, by definition, defined comparison groups retrospectively by exposure [24–26, 41]. An interrupted time series and before-after evaluation did not include comparison groups, but measured changes among those assigned or exposed to the intervention [32, 39]. No articles reporting non-RCT study designs described a sample size calculation to ensure sufficient statistical power [24–26, 32, 36, 39, 41], although all studies, except one [24], clearly described equivalent eligibility criteria in intervention and comparison groups. Studies without comparison groups did not control statistically for confounding [32, 39]; in one article, this appears to be consistent with the stated intention to conduct a proof of concept study, rather than an impact evaluation [32]. Two studies similarly did not report each outcome described in the methods among the results [39, 41], and none of the authors clearly stated that implementation and analyses were conducted independently of funders.

Across study designs, many articles did not report adherence to IPV research recommendations fully. Among 16 articles that measured IPV, four did not report restricting IPV questions to persons who are at least 15 years old [27, 29, 35, 37]; eight did not clearly report IPV-specific training for interviewers [24, 25, 29, 31, 32, 35, 39, 40]; and 10 did not report developing referral information or protocols to provide support for persons disclosing IPV [24, 25, 27, 29, 31, 32, 34, 37, 39, 40]. The extent to which reporting limitations suggest intervention or study limitations, however, is unknown across all measures of bias.

## Discussion

As the first study to systematically review the impact of structural interventions for male-perpetrated IPV in LMIC, several important findings emerge. First, the reviewed studies suggest the potential for structural interventions to reduce or prevent IPV in these settings. Social, economic, and combined economic and social interventions were associated with positive outcomes. Economic interventions demonstrated decreased odds of controlling behaviors [27, 29], improved economic wellbeing [27], and enhanced relationship quality [27]. Social interventions found reduced physical [42], psychological [39, 42], sexual [42], and physical or psychological IPV [39], in addition to support for more equitable gender norms [26, 36, 39, 40], reduced acceptance of IPV [41], enhanced relationship quality or male household participation [30, 35], improved help seeking [41], and greater collection action [41]. Combined economic and social interventions were associated with reduced IPV [28, 32–34, 37], improved economic wellbeing [32–34, 37], reduced acceptance of IPV [28, 34], more equitable gender norms [32], and a range of social outcomes reflecting relationship quality, empowerment, social capital, and collective action [27, 32–34, 37].

These changes occurred in relatively short time periods; only one study [6, 35] had a measured follow up period longer than two years. Whereas comparison of effect sizes is difficult due to heterogeneous effect size estimation, it is notable that effect sizes were large in some cases, including 61 % reduction in odds of past-year economic abuse in Côte d’Ivoire [28] and 55 % reduction in the risk of past-year physical or sexual IPV in IMAGE [33, 37]. Interventions also demonstrated the potential to transform social norms broadly, including affecting gender norms among potentially unexposed men in study areas of North India [26] and relationship quality or equity across study areas of urban Uganda [6, 35]. Further, per-protocol analyses demonstrated additional effects in some cases, such as increased consumption of nondurable goods by women and greater business-related partner support in Women Plus [27] and decreased physical IPV in Côte d’Ivoire [28]. Although we necessarily privileged intention to treat analyses, as issues related to intervention uptake and participation may result in significant differences between study and general populations, these results suggest additional promise. Incremental social norm change may support increased participation in these interventions over time, increasing their effectiveness.

These findings, however, should be understood in light of varied risk of bias. Three interventions failed to demonstrate statistically significant effects at *p* < 0.05 for outcomes of interest [24, 25, 31], and nearly all others demonstrated heterogeneous effects across indicators for at least one outcome of interest. The relative contribution of intervention design, context, and research methodology to this heterogeneity largely is unknown [7]. Of note, only four articles reporting RCTs [6, 27, 28, 42] described calculating and enrolling a sufficiently large sample to support statistical inference, suggesting the possibility for undetected intervention effects. Measurement of primary outcomes also demonstrated significant heterogeneity, including single and combined types of IPV experience and diverse recall periods, with limited contextual validation of study questions. There remains a need to develop measures that are comparable across contexts and empirically valid for the contexts to which they are applied in order to ensure meaningful, comparable effects.

Organizing studies through the framework of structural interventions suggests specific opportunities to broaden knowledge of prevention approaches in LMIC. Reviewed approaches included microfinance, cash transfers, livelihoods training, couples-focused education, participatory learning and community mobilization, educational entertainment, and combinations of these economic and social approaches. Robust evaluations of politico-legal interventions, such as legislation on ownership of economic assets, inheritance, and access to divorce for women, and emerging physical interventions (e.g., SafetiPin in India, a social media application that aggregates information about neighborhood safety) are needed. In particular, understanding the longer-term impact of first generation politico-legal interventions and evaluating these policies in light of shifting gender norms and current prevention strategies are important goals that require multidisciplinary collaboration.

These findings also suggest the need to evaluate intervention effects rigorously and disseminate findings in the peer-reviewed literature. Although the data did not permit quantitative evaluation of publication bias, screening records for this study and reviewing the growing IPV intervention database [43] suggested that *evaluations* of IPV interventions disproportionately occur in high-income countries, with relatively less evaluation and peer-reviewed dissemination for interventions conducted in LMIC. Multiple intervention strategies from LMIC are absent from this review, such as Horizons, which aimed to promote gender-equitable attitudes in Brazil, Ethiopia, India, Nicaragua, Tanzania, and Zimbabwe [44], and the Gender Equity Movement in Schools (GEMS), which presently aims to change social norms among young adolescents in India and Vietnam [45].

These findings further suggest that granting attention to structural IPV interventions requires thoughtful consideration of research methodology. Whereas it previously appeared that the high cost of community RCTs might limit application of this research design to structural interventions generally, this review notably includes nine cluster RCTs. This number grew exponentially during manuscript preparation, with twice as many published during 2013–2015 as 2000–2013. These studies, however, frequently encountered inadequate power to detect statistical significance. Multiple authors advocated for use of directionality, consistency, coherence, and plausibility of effect estimates as benchmarks for intervention success [6, 34]. By contrast, we privileged conventional benchmarks of statistical significance in this review and underscore the need for adequately powered evaluations, including an appropriate number of clusters. This is essential to identifying and comparing treatment effects, particularly as the growing body of evidence demonstrates the feasibility and plausibility of structural interventions.

Further, measurement of *structural* level impact was sparse. Approaches included cluster level analyses of longitudinal data from individuals enrolled in intervention and control conditions [30, 34, 37] and cluster level analyses of cross-sectional data from multiple independent, representative samples [6, 35]. Few studies considered the aggregate effects of IPV prevention interventions or potential synergies between structural and individual or dyadic interventions. Considering interactions between interventions and incremental effects of interventions on the broader social environment is important theoretically and empirically; prior studies have concluded effective interventions engage multiple stakeholders through varied mechanisms [3], and multiple social science theories suggest interventions may exert effects cumulatively. Simultaneously, studies must protect participant safety through increased use of IPV specific training and referral protocols for appropriate interventions and psychosocial support for women disclosing IPV [23].

Previous discussions of structural interventions have described challenges inherent to evaluating such interventions and have argued to adopt novel intervention designs and measures that can evaluate the impact of interventions on both complex causal pathways and distal outcomes [7]. Inclusion of intermediate (e.g., endorsement of more equitable gender norms) and distal outcomes (i.e., IPV) in this review reinforces this recommendation. For IPV, research further must contend with risk pathways that are contextually dependent and often incompletely understood. For example, theory and empirical research continue to grapple with conflicting evidence on the relationship between economic empowerment and IPV. Theories alternately predict that economic empowerment *diminishes* risk by improving the status of women in their households and increasing the viability of marital exit when violence exceeds acceptable levels or *increases* risk by raising the likelihood that men will use violence to establish and maintain inequitable relationships, particularly in instances where empowerment challenges inequitable gender norms held by male partners or community members [29, 46]. Understanding these circumstances and pathways is essential to ensuring empowerment interventions reduce IPV risk [46], and addressing theoretical and empirical issues related to the selection and operationalization of secondary or intermediate outcomes is imperative. Likely a combination of classically rigorous and novel intervention designs and measures is required.

### Limitations

As the first review to apply the concept of structural interventions to IPV prevention, this review is subject to limitations arising from the challenge of consistently and rigorously defining structural interventions and differentiating them from non-structural approaches. We selected comprehensive search terms and applied definitions consistently; however, more work is needed to develop the concept of structural interventions in the IPV literature. Similarly, although we used a combination of search strategies to capture relevant literature, our search may have omitted relevant studies. Other studies were omitted because we focused on the peer-reviewed literature, as noted previously. Further, although focusing on quantitative measures increased comparability across studies, sufficient data are not available yet for quantitative synthesis or to compare effectiveness by intervention domain, context, or population, including addressing competing theories of economic empowerment and IPV. Understanding “what works, for whom, and in what situations” additionally likely requires varied evaluation strategies [7], including qualitative and sub-sample analyses that were beyond the scope of this review. Answering questions about research methodology is imperative both to understanding the limitations of this review and the impact of structural interventions for IPV broadly.

## Conclusions

Although IPV prevention programming remains nascent in LMIC, this review demonstrates promising growth. Among 20 identified articles measuring the impact of structural interventions for male-perpetrated IPV in LMIC, we identified 10 articles published during 2000–2013 and an additional 10 articles published in the last two years. This growth underscores the need to organize evidence for IPV prevention in LMIC in meaningful ways, which reflect how inequities throughout the social ecology affect IPV risk and which motivate diverse approaches to IPV prevention.

This review suggests prioritizing structural approaches to developing and evaluating IPV prevention programming in LMIC. Our findings provide preliminary evidence that approaches addressing social or economic risk can reduce IPV and controlling behaviors, improve economic wellbeing, enhance relationship quality, increase empowerment and social capital, motivate new help-seeking behaviors and collective action, diminish social acceptability of IPV, and produce more equitable gender norms. Positive associations were found at multiple levels of the social ecology, suggesting structural interventions might interact synergistically with individual and dyadic interventions, which represent essential complementary approaches.

Positive associations, however, were not uniform across studies or indicators for most outcomes of interest. Three studies did not report statistically significant associations for any outcomes of interest. Given that the effect of contextual and methodological heterogeneity largely is unknown, further research is needed. Methodologically, evaluating the impact of structural interventions for IPV requires continued research on the range of proximate influences and causal pathways through which structural interventions influence IPV in varied contexts and development of indicators for these influences and pathways that are contextually valid and comparable across contexts. Evaluating the impact of structural interventions also requires rigorous, adequately powered interventions that measure effects across different levels of the social ecology, balanced with novel intervention designs. Publication of positive and null findings from these studies in the peer-reviewed literature is essential. Conceptually, evaluation of more diverse intervention approaches is imperative, as identified studies concentrate evidence at the intersection of social and economic interventions. Multidisciplinary collaboration that engages researchers not traditionally engaged in IPV research is important in this regard. Simultaneously, researchers must be aware of socio-demographic groups vulnerable to heterogeneous effects and unintended harms and adhere to recommendations for the safe conduct of IPV research.

Overall, this review uniquely consolidates available evidence for structural interventions. Although many recommendations are consistent with the call to action issued by Ellsberg and colleagues [3], the review also points to the potential for structural interventions to impact IPV in LMIC and highlights the way that this framework can improve efforts to prevent IPV in LMIC, especially by enhancing the conceptual diversity and methodological rigor of evaluated interventions.

## Additional files

Additional file 1: Table S1. PRISMA checklist. (PDF 87 kb) Additional file 2: Table S2. Characteristics of interventions and studies. (PDF 191 kb) Additional file 3: Table S3. Analyses of primary outcomes. (PDF 151 kb)

## References

[1] World Health Organization (2013). Global and regional estimates of violence against women: prevalence and health effects of intimate partner violence and nonpartner sexual violence.

[2] Michau L, Horn J, Bank A, Dutt M, Zimmerman C. Prevention of violence against women and girls: lessons from practice. Lancet. 2014; doi:10.1016/S0140-6736(14)61797-9. 10.1016/S0140-6736(14)61797-9 25467577

[3] Ellsberg M, Arango DJ, Morton M, Gennari F, Kiplesund S, Contreras M, et al. Prevention of violence against women and girls: what does the evidence say? Lancet. 2014; doi:10.1016/S0140-6736(14)61703-7. 10.1016/S0140-6736(14)61703-7 25467575

[4] Capaldi DM, Langhinrichsen-Rohling J (2012). Informing intimate partner violence prevention efforts: dyadic, developmental, and contextual considerations. Prev Sci.

[5] Jewkes R, Flood M, Lang J. From work with men and boys to changes of social norms and reduction of inequities in gender relations: a conceptual shift in prevention of violence against women and girls. Lancet. 2014; doi:10.1016/S0140-6736(14)61683-4. 10.1016/S0140-6736(14)61683-4 25467578

[6] Abramsky T, Devries K, Kiss L, Nakuti J, Kyegombe N, Starmann E (2014). Findings from the SASA! Study: a cluster randomized controlled trial to assess the impact of a community mobilization intervention to prevent violence against women and reduce HIV risk in Kampala, Uganda. BMC Med.

[7] Gupta GR, Parkhurst JO, Ogden JA, Aggleton P, Mahal A (2008). Structural approaches to HIV prevention. Lancet.

[8] Morrison A, Ellsberg M, Bott S (2007). Addressing gender-based violence: a critical review of interventions. World Bank Res Obs.

[9] Bott S, Morrison A, Ellsberg M (2005). Preventing and responding to gender-based violence in middle and lowincome countries: a global review and analysis.

[10] Heise L (2011). What works to prevent partner violence? An evidence overview.

[11] Blankenship KM, Friedman SR, Dworkin S, Mantell JE (2006). Structural interventions: concepts, challenges and opportunities for research. J Urban Health.

[12] International Association for Suicide Prevention: IASP special interest group on the prevention of intentional pesticide poisoning**.** http://www.iasp.info/prevention_of_intentional_pesticide_poisoning.php (2015). Accessed 2 Feb 2015.

[13] World Health Organization (2005). WHO multi-country study on women's health and domestic violence against women: summary report of initial results on prevalence, health outcomes, and women's responses.

[14] Temmerman M. Research priorities to address violence against women and girls. Lancet. 2014; doi:0.1016/S0140-6736(14)61840-7. 10.1016/S0140-6736(14)61840-7 25467581

[15] Moher D, Liberati A, Tetzlaff J, Altman DG (2009). Preferred reporting items for systematic reviews and meta-analyses: the PRISMA statement. PLoS Med.

[16] Ramsay J, Carter Y, Davidson L, Dunne D, Eldridge S, Feder G, et al. Advocacy interventions to reduce or eliminate violence and promote the physical and psychosocial well-being of women who experience intimate partner abuse. Cochrane Database Syst Rev. 2009; doi:10.1002/14651858.CD005043.pub2. 10.1002/14651858.CD005043.pub2 19588364

[17] Wathen C, MacMillan HL (2003). Interventions for violence against women: scientific review. JAMA.

[18] Bates LM, Hankivsky O, Springer KW (2009). Gender and health inequities: a comment on the Final Report of the WHO Commission on the Social Determinants of Health. Soc Sci Med.

[19] Angelucci M (2008). Love on the rocks: domestic violence and alcohol abuse in rural Mexico. B E J Econom Anal Policy.

[20] Krishnan S, Vohra D, de Walque D, Medlin C, Nathan R, Dow WH (2012). Tanzanian couples' perspectives on gender equity, relationship power, and intimate partner violence: findings from the RESPECT Study. AIDS Res Treat.

[21] Liberati A, Altman DG, Tetzlaff J, Mulrow C, Gotzsche PC, Ioannidis JP (2009). The PRISMA statement for reporting systematic reviews and meta-analyses of studies that evaluate health care interventions: explanation and elaboration. Ann Intern Med.

[22] Cochrane Collaboration. Cochrane handbook for systematic reviews of interventions. Higgins JPT, Green S, editors. 2011. http://handbook.cochrane.org. Accessed 16 Jan 2013.

[23] Ellsberg M, Heise L (2005). Researching violence against women: a practical guide for researchers and activists.

[24] Ahmed SM (2005). Intimate partner violence against women: experiences from a woman-focused development programme in Matlab. Bangladesh J Health Popul Nutr.

[25] Bobonis GJ, Gonzalez-Brenes M, Castro R (2013). Public transfers and domestic violence: the roles of private information and spousal control. Am Econ J Econ Policy.

[26] Das A, Mogford E, Singh SK, Barbhuiya RA, Chandra S, Wahl R (2012). Reviewing responsibilities and renewing relationships: an intervention with men on violence against women in India. Cult Health Sex.

[27] Green EP, Blattman C, Jamison J, Annan J (2015). Women's entrepreneurship and intimate partner violence: a cluster randomized trial of microenterprise assistance and partner participation in post-conflict Uganda. Soc Sci Med.

[28] Gupta J, Falb KL, Lehmann H, Kpebo D, Xuan Z, Hossain M (2013). Gender norms and economic empowerment intervention to reduce intimate partner violence against women in rural Côte d’Ivoire: a randomized controlled pilot study. BMC Int Health Hum Rights.

[29] Hidrobo M, Fernald L (2013). Cash transfers and domestic violence. J Health Econ.

[30] Hossain M, Zimmerman C, Kiss L, Abramsky T, Kone D, Bakayoko-Topolska M (2014). Working with men to prevent intimate partner violence in a conflict-affected setting: a pilot cluster randomized controlled trial in rural Côte d’Ivoire. BMC Public Health.

[31] Jewkes R, Nduna M, Levin J, Jama N, Dunkle K, Puren A (2008). Impact of Stepping Stones on incidence of HIV and HSV-2 and sexual behaviour in rural South Africa: cluster randomised controlled trial. BMJ.

[32] Jewkes R, Gibbs A, Jama-Shai N, Willan S, Misselhorn A, Mushinga M (2014). Stepping Stones and Creating Futures intervention: shortened interrupted time series evaluation of a behavioural and structural health promotion and violence prevention intervention for young people in informal settlements in Durban. South Africa BMC Public Health.

[33] Kim JC, Watts CH, Hargreaves JR, Ndhlovu LX, Phetla G, Morison LA (2007). Understanding the impact of a microfinance-based intervention on women's empowerment and the reduction of intimate partner violence in South Africa. Am J Public Health.

[34] Kim J, Ferrari G, Abramsky T, Watts C, Hargreaves J, Morison L (2009). Assessing the incremental effects of combining economic and health interventions: the IMAGE study in South Africa. Bull World Health Organ.

[35] Kyegombe N, Abramsky T, Devries KM, Starmann E, Michau L, Nakuti J (2014). The impact of SASA!, a community mobilization intervention, on reported HIV-related risk behaviours and relationship dynamics in Kampala. Uganda J Int AIDS Soc.

[36] Miller E, Das M, Tancredi DJ, McCauley HL, Virata MC, Nettiksimmons J (2014). Evaluation of a gender-based violence prevention program for student athletes in Mumbai. India J Interpers Violence.

[37] Pronyk PM, Hargreaves JR, Kim JC, Morison LA, Phetla G, Watts C (2006). Effect of a structural intervention for the prevention of intimate-partner violence and HIV in rural South Africa: a cluster randomised trial. Lancet.

[38] Pronyk PM, Harpham T, Busza J, Phetla G, Morison LA, Hargreaves JR (2008). Can social capital be intentionally generated? a randomized trial from rural South Africa. Soc Sci Med.

[39] Pulerwitz J, Jui W, Arney J, Scott LM (2015). Changing gender norms and reducing HIV and violence risk among workers and students in China. J Health Commun.

[40] Pulerwitz J, Hughes L, Mehta M, Kidanu A, Verani F, Tewolde S (2015). Changing gender norms and reducing intimate partner violence: results from a quasi-experimental intervention study with young men in Ethiopia. Am J Public Health.

[41] Usdin S, Scheepers E, Goldstein S, Japhet G (2005). Achieving social change on gender-based violence: a report on the impact evaluation of Soul City's fourth series. Soc Sci Med.

[42] Wagman JA, Gray RH, Campbell JC, Thoma M, Ndyanabo A, Ssekasanvu J (2015). Effectiveness of an integrated intimate partner violence and HIV prevention intervention in Rakai, Uganda: analysis of an intervention in an existing cluster randomised cohort. Lancet Glob Health.

[43] Liverpool John Moores University Center for Public Health, World Health Organization, Centers for Disease Control and Prevention: Violence prevention evidence base http://www.preventviolence.info/EvidenceBase (2014). Accessed 20 Dec 2014.

[44] Pulerwitz J, Michaelis A, Verma R, Weiss E (2010). Addressing gender dynamics and engaging men in HIV programs: lessons learned from Horizons research. Public Health Rep.

[45] International Center for Research on Women: Gender Equity Movement in Schools. http://www.icrw.org/where-we-work/gender-equity-movement-schools-gems (2015). Accessed 11 Jan 2015.

[46] Vyas S, Watts C (2009). How does economic empowerment affect women's risk of intimate partner violence in low and middle income countries? a systematic review of published evidence. J Int Dev.

[47] Heise LL (1998). Violence against women: an integrated, ecological framework. Violence Against Women.

[48] Dahlberg LL (1998). Youth violence in the United States: major trends, risk factors, and prevention approaches. Am J Prev Med.

[49] Jewkes R (2002). Intimate partner violence: causes and prevention. Lancet.

[50] Mosavel M, Ahmed R, Simon C (2011). Perceptions of gender-based violence among South African youth: implications for health promotion interventions. Health Promot Int.

[51] Roman CG, Reid SE (2012). Assessing the relationship between alcohol outlets and domestic violence: routine activities and the neighborhood environment. Violence Vict.

[52] McKinney CM, Caetano R (2009). Alcohol availability and intimate partner violence among US couples. Alcohol Clin Exp Res.

[53] Curandi CB, Mair C, Ponicki W, Remer L (2011). Alcohol outlets, neighborhood characteristics, and intimate partner violence: ecological analysis of a California city. J Urban Health.

[54] Banerjee PR (2014). Dowry in 21st-century India: the sociocultural face of exploitation. Trauma Violence Abuse.

[55] Fawole OI (2008). Economic violence to women and girls: is it receiving the necessary attention?. Trauma Violence Abuse.

[56] Gover A, Paul DP, Dodge M (2011). Law enforcement officers' attitudes about domestic violence. Violence Against Women.

[57] Dixon L, Graham-Kevan N (2011). Understanding the nature and etiology of intimate partner violence and implications for practice and policy. Clin Psychol Rev.

[58] Jones JH, Ferguson B (2009). Demographic and social predictors of intimate partner violence in Colombia: a dyadic power perspective. Hum Nat.

[59] Pick S, Contreras C, Barker-Aguilar A (2006). Violence against women in Mexico: conceptualization and program application. Ann N Y Acad Sci.

[60] Seifarth JE, McGowan CL, Milne KJ (2012). Sex and life expectancy. Gend Med.

[61] Connell R (2012). Gender, health and theory: conceptualizing the issue, in local and world perspective. Soc Sci Med.

[62] Krug EG, Mercy JA, Dahlberg LL, Zwi AB (2002). The world report on violence and health. Lancet.

