# Supplementary material for: Systematic review of structural interventions for intimate partner violence in low- and middle-income countries: organizing evidence for prevention
Source: BMC Public Health. 2015 Nov 23;15:1165. doi: 10.1186/s12889-015-2460-4 (PMC4657265; doi:10.1186/s12889-015-2460-4)
Supplement: Additional file 2: Table S2. — Characteristics of interventions and studies. (PDF 191 kb) [file 12889_2015_2460_MOESM2_ESM.pdf]

Table S2 Characteristics of interventions and studies

First author (year)	Category[1]	Country (setting)	Intervention				Study					
			Intervention arm	Comparison arm	Allocation / targeting	Exposure period	Data source (year)	Study design	Sample size[2]	Intervention sample	Comparison sample	Follow-up[3]
Abramsky (2014) [6]	S	Uganda (urban)	SASA!: community mobilization focused on power inequities, targeting men and women in varied roles (e.g., community activists [CA], leaders, police, and healthcare workers)	Community mobilization of police and healthcare workers only	Men and women in 8 purposively selected, pair-matched, randomly allocated communities	2007-2012 (2.8 years within this period due to political disruption)	Study-specific survey (2007, 2012)	Cluster RCT, multistage stratified random sampling of exposed population[4]	8 clusters (241-928)	Men or women (same gender as CA), 18-49 years, close proximity to CA, usual resident of household and shared food, community resident ≥1 year (partnered women for IPV and women reporting IPV for IPV response analyses)	Men or women (same gender as passive volunteer), 18-49 years, close proximity to passive volunteer, usual resident of household and shared food, community resident ≥1 year (partnered women for IPV and women reporting IPV for IPV response analyses)	4 years
Ahmed (2005) [24]	E	Bangladesh (rural)	BRAC: women-focused development (e.g., non-formal education, skill training, and collateral-free loans)	None	Poor households (<0.5 acres of land and sell manual labor for 100+ days annually) in Matlab HDSS areas	1992-1999[5]	BRAC-ICDDR,B survey (1999)	Cross-sectional, unspecified sampling	2044[6]	Women, married, 15-49 years, BRAC member households in BRAC-ICDDR,B study villages	Women, married, 15-49 years, low-income, non-BRAC member households in BRAC ICDDR,B study villages	N/A

Bobonis (2013) [25]	E	Mexico (rural)[7]	Oportunidades[8]: conditional cash transfers for mothers	None	Poor households (based on proxy-means test) in eligible localities (high marginality index and access to a primary and secondary school)	1997-2003[5]	National Survey on Relationships within the Household (ENDIREH 2003)	Cross-sectional, nationally representative, random sampling	2867	Women, ≥25 years, married or partnered since 1997 (intact households), children ≤11 years in 1997, receives government support benefits	Women, ≥25 years, married or partnered since 1997 (intact households), children ≤11 years in 1997, does not receive government support benefits	N/A
Das (2012) [26]	S	India (mostly rural)	Men's Action to Stop Violence Against Women (MASVAW): individual and community action by men	None	Men in Uttar Pradesh	2002–2009[5]	Study-specific survey (2009)	Cross-sectional, multi-level random, purposive, and convenience sampling	361-366	Men, MASVAW activists	Men, non-activists residing in villages with MASVAW activities ("influenced") and men residing in villages without MASVAW activities ("control")	N/A

Green (2015) [27]	E<br><br>E, S	Uganda (rural)	Trial 1: Women's Income Generating Support (WINGS): cash transfer and microenterprise training for women<br><br>Trial 2: Women Plus (W+): WINGS and gender/couples training for women and household decision-makers	Trial 1: none<br><br>Trial 2: WINGS	Vulnerable individuals nominated by community and selected by implementing organization in 120 purposively selected villages	Trial 1: 2009-2011<br><br>Trial 2: 2011-2012	Study-specific survey (2009, 2011, 2012)	Cluster RCT	Trial 1: 1734 (men and women), 1488 (women)<br><br>Trial 2: 749 (women)	Men and women, (privileging poor women, 14-30 years) residing in treatment village (women for IPV analyses)	Men and women, (privileging poor women, 14-30 years) residing in comparison village (women for IPV analyses)	Trial 1: 16 months<br><br>Trial 2: 1 year
Gupta (2013) [28]	E, S	Côte d'Ivoire (rural)	Village Savings and Loans Association (VSLA) for women and Gender Dialogue Groups (GDG) for VSLA members and male partners (or male family members if unpartnered)	VSLA	Women in 24 purposively selected villages	2010-2012 (8 sessions over 16 weeks beginning in 2011 for GDG)	Study-specific survey (2010, 2012)	Cluster RCT	913-934	Women and male partners or family members, ≥18 years, no prior group savings participation (partnered women for IPV analyses)	Women, ≥18 years, no prior group savings participation (partnered women for IPV analyses)	2 years (VSLA)<br><br>1 year (GDG)
Hidrobo (2013) [29]	E	Ecuador (mixed)	Bono de Desarrallo Humano (BDH): unconditional cash transfers for mothers	None	Poor households (2 lowest wealth quintiles) in randomly selected parishes from 6 provinces	2004-2006[5]	Study-specific survey (2003-2004 and 2005-2006)	Cluster RCT, random sampling of survey households	1218-1246	Women, living with spouse or partner, at least one preschool age child, no child >6 years, eligible for BDH, not recipient	Women, living with partner, at least one preschool age child, no child >6 years, eligible for BDH, not recipient of previous welfare	2 years

										of previous welfare program, intervention parish resident	program, control parish resident	
Hossain (2014) [30]	S	Côte d'Ivoire (rural)	Men's discussion group and community-based gender-based violence (GBV) prevention programming	Community-based GBV prevention programming	Men in 12 purposively selected, pair-matched, randomly allocated communities	2010-2011 (16 sessions over 4 months)	Study-specific survey (2010, 2012)	Cluster RCT	12 clusters (261-346)	Men, ≥15 years, intervention community resident (current female partner interviewed for analysis)	Men, ≥15 years, group age-matched to intervention village pair men, control community resident with exposure to community GBV programming (current female partner interviewed for analysis)	16 months
Jewkes (2008) [31]	S	South Africa (rural)	Stepping Stones (2nd edition): participatory gender and sexual health learning program targeting men and women	Brief intervention on HIV, safer sex, and condoms	Young men and women in randomly allocated villages (10 km from nearest program community, junior or senior secondary school, community willing to participate)	2003-2004 (50 hours over 6-8 weeks)	Study-specific survey (2003-2004, 2004-2005, 2005-2006)	Cluster RCT	537-559 (women), 504-534 (men)	Men and women, 15-26 years, normally reside in the village where they were at school	Men and women, 15-26 years, normally reside in the village where they were at school	1 year and 2 years

Jewkes (2014) [32]	E, S	South Africa (urban)	Participatory livelihoods training (Creating Futures) and Stepping Stones (3rd edition)	N/A	Young men and women in 2 informal settlements	Year not reported (33 hours over 12 weeks)	Study-specific survey	Time series	122 (women), 110 (men)	Men and women, 18-34 years, out of school	N/A	28 and 58 weeks
Kim (2007) [33]	E, S	South Africa (rural)	Intervention with Microfinance for AIDS and Gender Equity (IMAGE): microfinance and participatory learning program targeting women (Phase 1) and broader community mobilization (Phase 2)	None	Poor households (based on participatory wealth-ranking) in 8 pair-matched, randomly allocated villages	2001-2005 (2 years during period)	Study-specific survey	Cluster RCT	361-845	Women, ≥18 years, applied for loan, intervention village resident	Women, ≥18 years, control village resident pair-matched to intervention participant	2 years
Kim (2009) [34]	E, S	South Africa (rural)	Intervention with Microfinance for AIDS and Gender Equity (IMAGE)	Microfinance only (MF only) or none	Poor households (based on participatory wealth-ranking) in 12 matched, randomly allocated villages	2001-2005 (2 years during period)	Study-specific survey	Cluster RCT	12 clusters (331-867)	Women, ≥18 years, applied for loan, intervention village resident (partnered women for IPV and controlling behavior analyses)	MF only: women, joined MF program in MF only village<br><br>Control: women, ≥18 years, control village resident pair-matched to intervention participant (partnered women for IPV and controlling	2 years

											behavior analyses)	
Kyegombe (2014) [35]	S	Uganda (urban)	SASA!: community mobilization focused on power inequities, targeting men and women in varied roles (e.g., community activists [CA], leaders, police, and healthcare workers)	Community mobilization of police and healthcare workers only[9]	Men and women in 8 pair-matched, randomly-allocated communities[9]	2007-2012 (2.8 years within this period due to political disruption)[9]	Study-specific survey (2007, 2012)	Cluster RCT, multistage stratified random sampling of exposed population[4,9]	8 clusters (495-776 women, 473-906 men)	Men or women (same gender as CA), 18-49 years, close proximity to CA, usual resident of household and shared food, community resident ≥1 year,[9] non-polygamous, regular partner in past year (women for IPV analyses)	Men or women (same gender as passive volunteer), 18-49 years, close proximity to passive volunteer, usual resident of household and shared food, community resident ≥1 year,[9] non-polygamous, regular partner in past year (women for IPV analyses)	4 years
Miller (2014) [36]	S	India (urban)	Parivartan: bystander intervention targeting boys	None	Adolescent boys on cricket teams in 46 purposively selected schools active in the Mumbai School Sports Association	Year not reported (4 months)	Study-specific survey	Non-randomized trial	309	Boys, 10-16 years, cricket team member in intervention school	Boys, 10-16 years, cricket team member in control school	1 year
Pronyk (2006) [37]	E, S	South Africa (rural)	Intervention with Microfinance for AIDS and Gender Equity (IMAGE)	None	Poor households (based on participatory wealth-ranking) in 8 pair-matched, randomly allocated villages	2001-2005 (2 years within this period)	Study-specific survey	Cluster RCT	8 clusters (524-2221)	Women, applied for loan, intervention village resident[10]	Women, control village resident pair-matched to intervention participant (randomly selected)	2 years

Pronyk (2008) [38]	E, S	South Africa (rural)	Intervention with Microfinance for AIDS and Gender Equity (IMAGE)	None	Poor households (based on participatory wealth-ranking) in 8 pair-matched, randomly allocated villages	2001-2005 (2 years within this period)	Study-specific survey	Cluster RCT	8 clusters (810-845)	Women, applied for loan, intervention village resident	Women, control village resident pair-matched to intervention participant	2 years
Pulerwitz (2015) [39]	S	China (not stated)	Participatory gender learning program targeting men	N/A	Young men from 3 vocational schools and 8 workplaces	2010 (eight 2-hour sessions over 6 months)	Study specific survey (2010, 2010)	Before-after evaluation	219 workers, 496 students	Men, 15-24 years, employed at selected workplaces or non-graduating students at vocational schools	N/A	9 months
Pulerwitz (2015) [40]	S	Ethiopia (urban)	Male Norms Initiative: interactive group education (GE) and community engagement (CE) targeting men	CE only or none	Young men from 11 youth groups in 3 sub-cities	June-Nov. 2008 (eight 2- or 3-hour sessions for GE)	Study-specific survey	Quasi-experimental cluster RCT	645[6]	Men, 15-24 years, member of youth group in intervention sub-city (partnered men for IPV analyses)	Men, 15-24 years, member of youth group in CE only or control sub-city (partnered men for IPV analyses)	6 months
Usdin (2005) [41]	S	South Africa (mixed)	Soul City Fourth Series (SC4): national domestic violence media campaign targeting men and women	None[11]	Men and women in "African" and "Colored" communities	July-Dec. 1999	Study-specific survey	Before-after evaluation, stratified national random sampling	1981[6]	Men and women, 16-65 years, "African" or "Colored"	N/A[11]	7 months

Wagman (2015) [42]	S	Uganda (rural)	Safe Homes and Respect for Everyone (SHARE): community mobilization and HIV intervention	Standard of care HIV services	Men and women from 11 randomly allocated clusters	2006-2009	Study-specific survey (2005-2006, 2006-2008, 2008-2009)	Cluster RCT	3775-4768 (women), 2511-3074 (men)	Men and women, 15-49 years (completed Rakai Community Cohort Study interview and provided blood for HIV testing at baseline and follow up for analyses)	Men and women, 15-49 years (completed Rakai Community Cohort Study interview and provided blood for HIV testing at baseline and follow up for analyses)	16 months, 35 months

[1]Abbreviations represent categories of structural interventions: E = economic and S = social.
[2]Sample sizes are reported for the most inclusive model(s) for outcome(s) of interest. For studies with multiple outcomes of interest, ranges reflect the models with the smallest and largest sample sizes. Inclusion of cases with missing data is assumed where authors did not specify complete case analysis.
[3]The length of follow-up is given as the time from intervention initiation to follow-up measurement.
[4]The study evaluation used cross-sectional surveys with independent samples at baseline and follow-up, representing the population "mostly likely to have had repeated and extensive contact with intervention activities" (p. 4) [6].
[5]The exposure period represents the period from intervention initiation to data collection, or the total potential exposure period. Exposure periods may differ among individual participants; the authors did not report measuring exposure periods for individual participants.
[6]The presented sample size represents the full study sample. The authors did not report sample size(s) for outcome(s) of interest.
[7]*Oportunidades* targeted rural, semi-urban, and urban localities. This represents an analytic restriction.
[8]The study measured participation in *Oportunidades* as "whether the woman receives benefits from any government support program" (p. 186) [26]. The authors note that "there are other small government programs that provide non-cash benefits," although "*Oportunidades* is the largest and most generous cash transfer program" (p. 186) [26].
[9]As described in Abramsky [6]
[10]The study included three cohorts. We present only cohort one, composed of IMAGE participants and matched controls, because measured outcomes in cohorts two and three focused on HIV.
[11]The study compares the full sample before and after the intervention. Groups also were defined retrospectively at follow-up by intervention exposure (no/low exposure, moderate exposure, and high media exposure).
