# Supplementary material for: Systematic review of structural interventions for intimate partner violence in low- and middle-income countries: organizing evidence for prevention
Source: BMC Public Health. 2015 Nov 23;15:1165. doi: 10.1186/s12889-015-2460-4 (PMC4657265; doi:10.1186/s12889-015-2460-4)
Supplement: Additional file 3: Table S3. — Analyses of primary outcomes. (PDF 151 kb) [file 12889_2015_2460_MOESM3_ESM.pdf]

Table S3 Analyses of primary outcomes

First author (year)	Statistical test	Effect measure	Primary outcome(s)[1]	Effect size	Adjustment factors
Abramsky (2014) [6]	ANOVA	Risk ratio (95% CI)	**IPV**: Physical violence in past year<br>**IPV**: Sexual violence in past year	0.48 (0.16-1.39)<br>0.76 (0.33-1.72)	Age, marital status, baseline area-level prevalence of IPV, community-pair
Ahmed (2005) [24]	Logistic regression	Odds ratio (95% CI)	**IPV**: Physical or psychological violence in past 4 months	Eligible non-member: 1.00<br>Passive member (savings): 1.36 (0.79-2.36)<br>Active member (savings + credit): 1.47 (0.93-2.33)<br>Skilled member (savings + credit + training): 0.64 (0.25-1.66)	Age, schooling, contribution to household income, currently-alive children, age of household head, schooling of household head, household poverty status
Bobonis (2013) [25]	OLS regression	Odds ratio,[3] *p*-value	**IPV**: Physical or sexual violence in past 12 months<br>**IPV**: Physical violence in past 12 months<br>**IPV**: Psychological violence in past 12 months<br>**IPV**: Threats of physical violence in past 12 months<br>**IPV**: Sexual violence in past 12 months	0.921, $p<0.1$<br>0.946, $p<0.1$<br>1.027, $p>0.1$<br>1.018, $p>0.1$<br>0.951, $p>0.1$	Woman and partner's age, woman and partner's indigenous status, woman's schooling, partner's schooling, household size, cohabitating couple indicator, years in union, spousal abuse exposure during childhood, individual control interaction terms, individual control polynomial terms, village fixed effects
Das (2012) [26]	OLS regression	Odds ratio,[3] *p*-value	Women's role/autonomy<br>Gender roles<br>Domestic work<br>Masculinity<br>Sexuality<br>Knowledge of women/child laws<br>Women do	Activist: 20.29, p=0.000<br>Influenced: 9.68, p=0.000<br>Activist: 132.95, p=0.000<br>Influenced: 24.29, p=0.000<br>Activist: 2.46, p=0.000<br>Influenced: 1.90, p=0.000<br>Activist: 26.31, p=0.000<br>Influenced: 6.89, p=0.000<br>Activist: 12.30, p=0.000<br>Influenced: 4.18, p=0.000<br>Activist: 3.13, p=0.002<br>Influenced: 0.83, p=0.532<br>Activist: 0.06, p=0.000	Age, education, caste, marital status, family structure, religion, occupation

			"traditional women's work"	Influenced: 0.21, *p*=0.000	
			Men do "traditional male work"	Activist: 0.08, *p*=0.000<br>Influenced: 0.24, *p*=0.000	
Green (2015) [27]	OLS regression	Odds ratio[3] (95% CI), *p*-value	**IPV**: Physical or psychological violence in past 8 months	Trial 1 (WINGS v. control): 1.02 (0.90-1.15), *p*>0.1<br>Trial 2 (W+ v. WINGS): 1.01 (0.87-1.17), *p*>0.1	District fixed effect, road distances between villages, 90 baseline and midline covariates
			**IPV**: Marital control in past 8 months	Trial 1 (WINGS v. control): 1.15 (1.01-1.31), *p*<0.05<br>Trial 2 (W+ v. WINGS): 0.99 (0.83-1.19), *p*>0.1	
Gupta (2013) [28]	GMM (4-level random intercepts model)	Odds ratio (95% CI), *p*-value	**IPV**: Physical or sexual violence in past year	0.92 (0.58-1.47), *p*=0.72	Cluster
			**IPV**: Physical violence in past year	0.69 (0.39-1.21), *p*=0.19	
			**IPV**: Sexual violence in past year	0.71 (0.40-1.25), *p*=0.24	
			**IPV**: Economic abuse in past year	0.39 (0.25-0.60), *p*<0.0001	
Hidrobo (2013) [29]	Linear probability model	Odds ratio,[3] *p*-value	**IPV**: Physical violence in lifetime	0.98, *p*>0.1	Woman's age, years of schooling, race (Afro-Ecuadorian or indigenous), marital status, partner's years of schooling, history of child death, current pregnancy, number of children 0-5 years old, urban indicator, use of kitchen for sleeping, asset index, asset index squared
			**IPV**: Psychological violence (phrased in the present)	0.98, *p*>0.1	
			**IPV**: Controlling behaviors (phrased in the present)	0.94, *p*<0.05	
Hossain (2014) [30]	Unpaired t-test	Risk ratio (95% CI)	**IPV**: Physical or sexual violence in past 12 months	0.52 (0.18-1.51)	Age group, cohabitation status, literacy, traumatic

			**IPV**: More than one act of physical violence or at least one severe act in past 12 months	0.64 (0.24-1.73)	experiences, baseline outcome level, village-pair
			**IPV**: Sexual violence in past 12 months	0.50 (0.14-1.80)	
Jewkes (2008) [31]	GLMM/GEE	Odds ratio (95% CI), *p*-value	**IPV**: More than 1 incident of physical or sexual violence since last interview (in past 12 months)	Women (receipt)<br>12 months: 0.87 (0.64-1.18), *p*=0.36<br>24 months: 1.14 (0.77-1.68), *p*=0.51<br>Men (perpetration)<br>12 months: 0.73 (0.50-1.06), *p*=0.099<br>24 months: 0.62 (0.38-1.01), *p*=0.054	Geographic stratum, age, time since last visit
Jewkes (2014) [32]	Random effects regression	Model determined trend, *p*-value	**IPV**: Physical or sexual violence in last 3 months	Women (receipt) at baseline, 2 weeks, 28 weeks, and 52 weeks: 30.3, 25.7, 27.4, 18.9, *p*=0.037<br>Men (perpetration) at baseline, 2 weeks, 28 weeks, and 52 weeks: 23.9, 25.3, 26.0, 21.9, *p*=0.86	None
			**IPV**: Physical violence in last 3 months	Women (receipt) at baseline, 2 weeks, 28 weeks, and 52 weeks: 27.9, 18.3, 25.6, 18.0, *p*=0.12<br>Men (perpetration) at baseline, 2 weeks, 28 weeks, and 52 weeks: 16.5, 16.5, 17.3, 12.5, *p*=0.49	
			**IPV**: Sexual violence in last 3 months	Women (receipt) at baseline, 2 weeks, 28 weeks, and 52 weeks: 9.8, 12.5, 7.7, 3.6, *p*=0.033<br>Men (perpetration) at baseline, 2 weeks, 28 weeks, and 52 weeks: 14.7, 16.5, 12.5, 13.5, *p*=0.69	
Kim (2007) [33]	Logistic regression	Risk ratio (95% CI)	**IPV**: Physical or sexual violence in past year	0.45 (0.23-0.91)	Age, village pair, marital status (only IPV and attitude measures), baseline measure, lifetime experience of IPV by current partner at baseline (only IPV measure)
			**IPV**: Controlling behavior in past year	0.80 (0.35-1.83)	

Kim (2009) [34]	ANOVA and logistic regression	Risk ratio (95% CI)	**IPV**: Physical or sexual violence in past year	MF[4] v. control: 0.86 (0.22-3.36)<br>IMAGE v. control: 0.51 (0.28-0.93)<br>IMAGE v. MF: 0.59 (0.09-3.66)	Age group, village triplet, marital status (only attitude measure, others restricted to currently partnered women), education, parity, sex of household head
			**IPV**: Controlling behavior in past year	MF v. control: 1.18 (0.77-1.80)<br>IMAGE v. control: 0.84 (0.38-1.87)<br>IMAGE v. MF: 0.69 (0.35-1.36)	
Kyegombe (2014) [35]	Geometric mean prevalence ratio (statistical test unspecified)	Risk ratio (95% CI)	**IPV**: Sexual violence in past year	0.81 (0.31-2.10)	Age, marital status, baseline area-level prevalence of the outcome, community-pair
Miller (2014) [36]	Linear mixed-effects regression	Between-group comparison on change score (95% CI), *p*-value	Gender attitudes	0.28 (0.12-0.43), *p*=0.001	Age group, religion, school
			Attitudes disapproving of violence against women	0.04 (-0.31-0.39), *p*=0.82	
Pronyk (2006) [37]	Logistic regression	Risk ratio (95% CI)	**IPV**: Physical or sexual violence in past year	0.45 (0.23-0.91)	Age, village pair, marital status (IPV and attitudes measure only), baseline measure, lifetime experience of IPV by current partner at baseline (IPV measure only)
			**IPV**: Controlling behavior in past year	0.80 (0.35-1.83)	
Pronyk (2008) [38]	Logistic regression	Risk ratio (95% CI)	More participation in social groups	1.85 (0.95-3.61)	Age, village pair, marital status, baseline measure
			Belief in community support towards common goals	1.11 (0.38-3.24)	
			Greater perception of community solidarity in a time of crisis	1.65 (0.81-3.37)	
			Taken part in collective action	2.06 (0.92-4.49)	

Pulerwitz (2015) [39]	Chi-square test or bivariate regression	Not reported, *p*-value	**IPV**: Physical or psychological violence in past 3 months	Workers at baseline v. follow-up: 25.00 v. 10.94, $p<0.05$<br>Students at baseline v. follow-up: 10.61 v. 3.16, $p<0.05$	None
			**IPV**: Physical violence in past 3 months (perpetration)	Workers at baseline v. follow-up: 19.23 v. 10.94, $p>0.05$<br>Students at baseline v. follow-up: 7.58 v. 3.16, $p>0.05$	
			**IPV**: Psychological violence in past 3 months (perpetration)	Workers at baseline v. follow-up: 11.54 v. 1.56, $p<0.05$<br>Students at baseline v. follow-up: 5.30 v. 1.58, $p<0.05$	
Pulerwitz (2015) [40]	GEE	Odds ratio, *p*-value	**IPV**: Physical or sexual violence in the last 6 months (perpetration)	GE+CE[5] v. control: 60% less likely, *p*-value not given<br>CE v. control: 60% less likely, *p*-value not given	Age, GEM Scale score, time, time by intervention group
			**IPV**: Physical, sexual, or psychological violence in the last 6 months (perpetration)	GE+CE v. control: 55% less likely, *p*-value not given<br>CE v. control: 65% less likely, $p=0.06$	
Usdin (2005) [41]	Logistic regression	Baseline to follow-up: percent difference, *p*-value[6]<br><br>Follow-up by exposure: within column percentages[6]	Knowledge and awareness	Baseline to follow-up: 14% increase, $p\leq0.05$<br>By exposure at follow-up: 4-61%[7]	Baseline to follow-up: area (urban or rural), age, sex, race, education, employment status, general media exposure<br><br>Follow-up by exposure: area (urban or rural), age, sex, race, education, employment status, general media exposure
			Personal attitudes	Baseline to follow-up: non-significant to 18% increase[7], $p\leq0.05$<br>By exposure at follow-up: 58-97%[7]	
			Subjective norms	Baseline to follow-up: 18-59% increase[7], $p\leq0.05$<br>By exposure at follow-up: no association to 90%[7]	
			Behaviors	Baseline to follow-up: not measured at baseline<br>By exposure at follow-up: 0-62%[7]	
Wagman (2015) [42]	Modified Poisson multivariate regression	Prevalence risk ratios (95% CI)	**IPV**: Physical violence in past year	Women (receipt)<br>16 months: 0.97 (0.85-1.11)<br>35 months: 0.79 (0.67-0.92)<br>Men (perpetration)<br>16 months: 0.80 (0.64-1.00)<br>35 months: 1.00 (0.77-1.30)	Age, education, marital status, baseline experience of IPV experience (women) or perpetration (men)
			**IPV**: Sexual violence in past year	Women (receipt)<br>16 months: 1.12 (0.96-1.31)<br>35 months: 0.80 (0.67-0.97)	

	Men (perpetration)<br>16 months: 0.90 (0.63-1.28)<br>35 months: 0.81 (0.52-1.26)
**IPV**:<br>Psychological violence in past year	Women (receipt)<br>16 months: 1.02 (0.92-1.14)<br>35 months: 0.91 (0.79-1.04)<br>Men (perpetration)<br>16 months: 0.88 (0.78-0.98)<br>35 months: 0.99 (0.85-1.16)

[1]Psychological IPV includes violence classified as psychological, emotional, and verbal by authors.
[2]Questions classified as psychological violence included controlling behaviors.
[3]We transformed reported beta-coefficients into odds ratios to increase comparability of effect sizes.
[4]MF = microfinance only intervention
[5]GE = group education, CE = community engagement
[6]Some measures were restricted to specific audiences (i.e., national, urban, or rural audiences or women) or specific media exposures (i.e., exposure to three media sources or high exposure to television, radio, or print media).
[7]The reported range is the smallest and largest percentage for all indicators in the stated category.
